# A Solution to the Common Problem of the Synthesis and Applications of Hexachlorofluorescein Labeled Oligonucleotides

**DOI:** 10.1371/journal.pone.0166911

**Published:** 2016-11-18

**Authors:** Andrey N. Chuvilin, Igor P. Smirnov, Alena G. Mosina, Anna M. Varizhuk, Galina E. Pozmogova

**Affiliations:** Department of Molecular Genesis, Federal Research and Clinical Center of Physical- Chemical Medicine, Moscow, Russian Federation; Helsingin Yliopisto, FINLAND

## Abstract

A common problem of the preparation of hexachlorofluorescein labeled oligonucleotides is the transformation of the fluorophore to an arylacridine derivative under standard ammonolysis conditions. We show here that the arylacridine byproduct with distinct optical characteristics cannot be efficiently separated from the major product by HPLC or electrophoretic methods, which hampers precise physicochemical experiments with the labeled oligonucleotides. Studies of the transformation mechanism allowed us to select optimal conditions for avoiding the side reaction. The novel method for the post-synthetic deblocking of hexachlorofluorescein-labeled oligodeoxyribonucleotides described in this paper prevents the formation of the arylacridine derivative, enhances the yield of target oligomers, and allows them to be proper real-time PCR probes.

## Introduction

Fluorophore-bearing oligodeoxyribonucleotides (ODNs) are widely used in molecular biology and medical diagnostics. Their applications as probes in real-time PCR (RTPCR) DNA analyses have been explicitly described [1]. Hexachlorofluorescein (HEX), a popular orange dye, is utilized as a simple fluorophore label for DNA probes [2, 3], for measuring changes in fluorescence [4, 5], or for other applications [6–11] where HEX-ODNs interact with biopolymers. HEX-ODNs are common probes for conventional PCR, as well as for quantitative and multiplex RTPCRs [12–18]. In particular, HEX label was used in so-called multicolor combinatorial probe coding, where one of 13–15 possible DNA targets is defined during a single PCR; the requisite number of probes is labeled with one or 2–4 different fluorophores, including HEX [19]. The presence of a definitive DNA binding site in the sample was confirmed from the shapes and proportions of four curves of fluorescence accumulation (CFAs).

The influence of fluorophores and quenchers (QU) on the stability of DNA duplexes and DNA probes has been investigated with carefully purified probes [20].

It is clear that for monitoring subtle differences in dye characteristics and for quantitative and multiplex bioassays, HEX-probes must be of special and constant purity.

S1 Table shows that many researchers purchase HEX-ODNs elsewhere and use them without any additional refinement or quality verification. However, the majority of ODN manufacturers guarantee a purity of only 85% for the probes.

**Fig 1:**
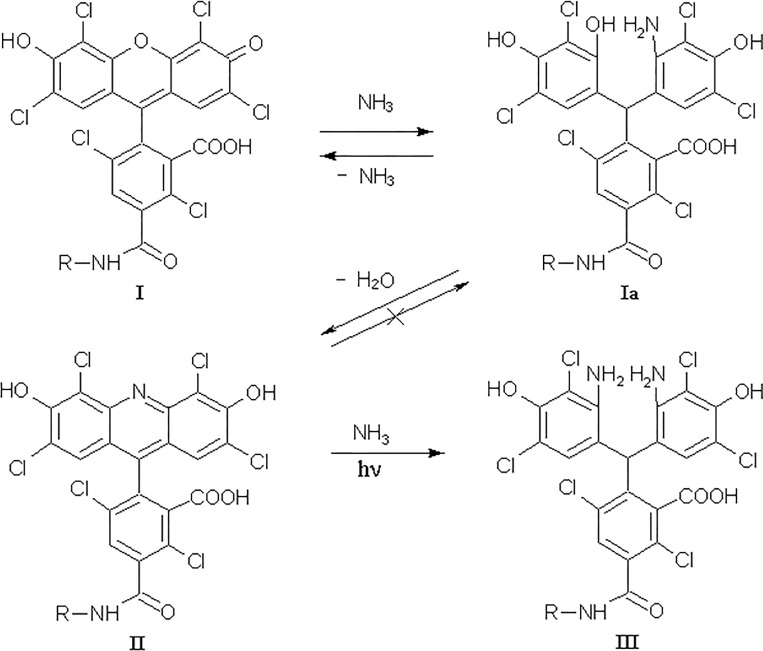
The transformation of hexachlorofluorescein dye (I) with aqueous ammonia and the next photochemical arylacridine transformation. R = 5'-oligodeoxyribonucleotide-spacer

We have shown earlier that HEX-labeled ODNs, even after optimized postsynthetic deblocking by concentrated aqueous ammonia, contain more than 25% of full-size ODN admixture. We discovered that during standard DNA deprotection, the HEX-residue (Fig 1, I) reacts with aqueous ammonia to give a hexachloroarylacridine (ACR) derivative (Fig 1, II), which has differed UV-VIS and fluorescence properties [21]. ACR impurities in HEX-ODNs may significantly distort optical and physicochemical characteristics of the probe and may lead to imprecise, inaccurate, or false results. In our previous work [22] we demonstrated that HEX-probes purified with a reverse phase (RP) cartridge or electrophoresis give inconsistent RTPCR results, and only RP HPLC could ensure the proper quality of HEX-probes and the reproducible accuracy of PCR. In those cases, when the HEX label is to be deblocked, it is necessary to achieve the smallest degree of modification.

We were surprised that prior to the completion of our study [21], the transformation of hexachlorofluorescein label with aqueous ammonia had not been described; as such, there were no recommendations for its prevention. In one study, wherein a new peak had been observed in an HPLC profile after ammonolysis of HEX-Dabsyl-ODN, the idea arose that this peak may have appeared due to degradation of the dye [23], without much further discussion or investigation ensuing. Unfortunately, the problem of acridine impurities present after hexachlorofluorescein-ODNs ammonolysis still has not been resolved.

## Materials and Methods

All ODNs were produced by a DNA synthesizer ASM 800 (Biosset Ltd., RF) in automatic mode. Standard nucleoside phosphoramidites, 5'-hexachlorofluorescein phosphoramidite (HEX-amidite), standard nucleoside and quenchers supports (3'-Dabsyl CPG, 3'-BHQ-1 CPG and 3'-BHQ-2 CPG) were purchased from Glen Research (Sterling, Virginia, USA). One synthetic column contained 9±0.5 mg of CPG. All interesting samples of synthesized ODNs are listed in S2 Table.

Real-time PCR was performed on an iCycler iQ5 (Bio-Rad, Hercules, California, USA) in PCR-buffer (pH 8.5 / 25°C, Lytech, RF) and was supplied with the proper quantities of DNA-target (10000 copies), nucleotide triphosphates, forward and reverse primers, and TAQ-polymerase. The curves of fluorescence accumulation (CFAs) were recorded at λ_ex_ = 530 nm and λ_em_ = 565 nm according to the following protocol:

**Table:** 

Cycle 1: (1X)		
Step 1:	94.0°C	for 01:30
Cycle 2: (40X)		
Step 1:	94.0°C	for 00:10
Step 2:	64.0°C	for 00:11
Data collection and real-time analysis enabled		
Step 3:	72.0°C	for 00:20

HPLC was performed on an Agilent Chemstation 1100 Series (Agilent Technologies, Santa Clara, California, USA) with Nucleosil 300 C18 or C4 columns (4.6x250 mm) (Macherey-Nagel, Germany), buffer "A" (0.1 M ammonium acetate in water (pH 6.7)) and buffer "B" (0.1 M ammonium acetate in 50% acetonitrile). The conditions of analytical HPLC were as follows: temperature 50°C, flow 0.85 ml/min; "18-H"-technique–C18 column, 20–50% "B"-buffer / 25 min; "18-HQ"-technique–C18 column, 30–60% "B"-buffer / 25 min; and "4-HQ"-technique–C4 column, 25–50% "B"-buffer / 25 min. Preparative HPLC gradients were individually chosen for each ODN.

Fluorescence spectra were recorded during HPLC: in 0.1 M NH_4_OAc at 45–50°C with the matrix detector Agilent 1100 Series G1321A; and in PCR-buffer at 64°C using a Chirascan Spectrofluorimeter (Applied Photophysics, UK).

UV-VIS spectra were obtained in water with an ND-1000 spectrophotometer (NanoDrop Technologies, USA) and during HPLC in 0.1 M NH_4_OAc using the matrix UV-detector Agilent 1100 Series G1315B.

Mass-spectra were recorded on MALDI-TOF mass-spectrometers UltraFlex and MicroFlex (Bruker, Germany) with the use of MSP target polished steel (Bruker, Germany) and the substrate 3-hydroxypicolic acid (Fluka, USA).

## Results and Discussion

**Fig 2:**
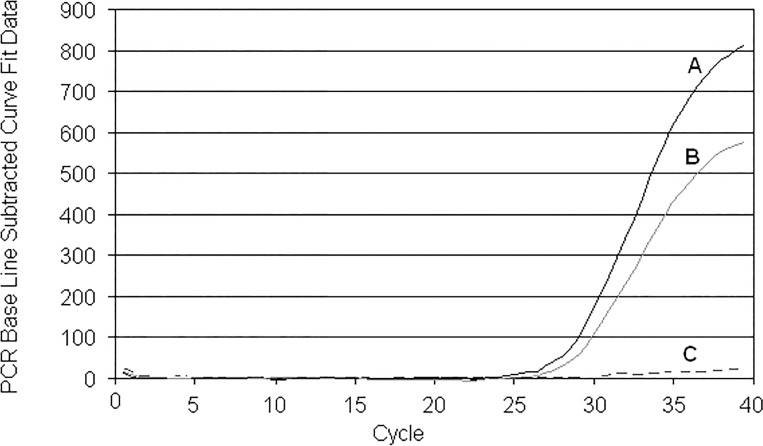
The curves of fluorescence accumulation (CFAs) obtained for H38B2 and A38B2 probes (see S2 Table). The curves obtained with: (A) pure probe, (B) artificial mixture (3:1, mol/mol) of H38B2 with its acridine analog A38B2, (C) pure A38B2. The amounts of amplicone were equal in every analysis, judjing by electrophoresis data as so as mentioned in [21].

When we discovered the phenomenon of hexachlorofluorescein (HEX) transformation to hexachloroarylacridine (ACR), we noted that reverse phase HPLC (RP HPLC) did not always give effective results following HEX-ODN purification. In some cases, peaks signifying ACR- and HEX-ODNs were hardly separated, even in analytical mode. We were also faced with a strange fact: substitution of HEX-probe with its ACR-analog led to ~ 40-fold decrease of the fluorescence signal during RTPCR (Fig 2, compare A and C curves). It became necessary i) to investigate the differences in the optical properties of the HEX probes and their ACR counterparts, ii) to compare their behavior during PCR and iii) to elaborate an alternative method of postsynthetic deprotection of HEX-ODNs that would ensure minimal quantities of ACR derivatives.

### Comparison of HEX and ACR optical properties

We compared the optical characteristics of HEX and ACR fluorophores (Table 1) from our earlier study [21] with HEX-T_10_ (HT10), its ACR-analog (AT10), and their mixtures at different molar ratios.

**Table 1:** Optical characteristics of HEX- and ACR-fluorophores.

Fluorophore, conditions (20–25°C)	VIS spectra		Fluorescence spectra		
	λ_max_, nm	ε at λ_max_, L/mol/cm	λ_max_ex, nm	λ_max_em, nm	Quantum yield
Hexachlorofluorescein	540±2	90000±1000	535±1	553±1	0.7
Hexachloroarylacridine	496±2	34000±500	495±1	537±1	0.1

The measurements were performed in H_2_O, in 0.1 M ammonium acetate, pH 6.7, 15–25%

MeCN, 40–50°C or in PCR-buffer at 64°C.

**Fig 3:**
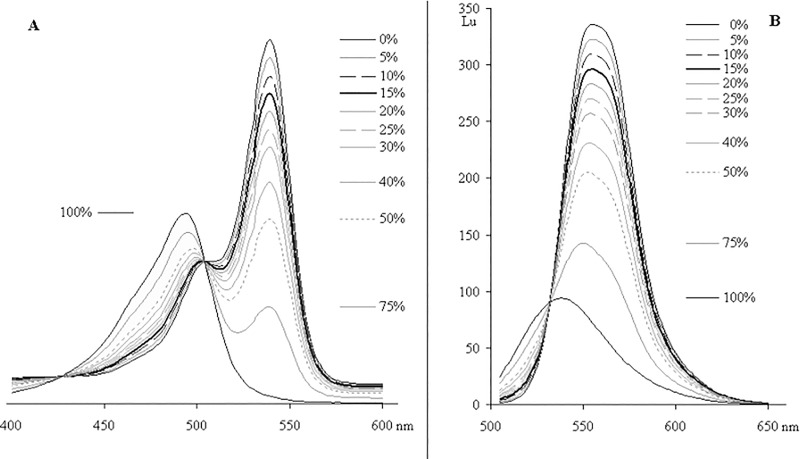
Spectra of mixtures of HEX-T_10_ (HT10) with different molar percentages of its arylhexachloroacridine analog (AT10). (A) VIS- and (B) fluorescent spectra in 0.1 M ammonium acetate, pH 6.7, 20% MeCN, 45°C. Baselines of spectra are moved apart to separate the curves.

We found that VIS-spectrum allows recognition of trace amounts of ACR admixture as low as 5–6%. At this value, the maximum in the curve, which is absent in the spectrum of pure HEX-ODN, appears at ~ 500 nm (Fig 3A). Fluorescence spectra (Fig 3B) are less informative and need to be compared with pure standards; this consideration is valid for any HEX-ODN unless it contains a quencher.

Real-time PCR probes always have some quencher. As a rule, one of the following quenchers (QU) is used with HEX-labeled ODNs: Black Hole 1 (BHQ1), Black Hole 2 (BHQ2, most frequent), or Dabsyl (Dabs). The fluorophore is attached to 5'-position and the quencher is located at 3'-end. We synthesized three model 5'-HEX-d(ACGT)_3_-derivatives with BHQ1- (H12B1 ODN), BHQ2- (H12B2) or Dabs-quencher (H12D) at the 3'-terminus.

H12B2 ODN had the sequence 5'-HEX-d((ACGT)_2_-ACG(G,T))-BHQ2-3' because some RTPCR probes may be mixed. Separation of such samples into individual compounds is not necessary, but it is impossible to predict, would the target HPLC band be single or double and would this (these) band(s) coincide or not with ACR admixture peak(s).

**Fig 4:**
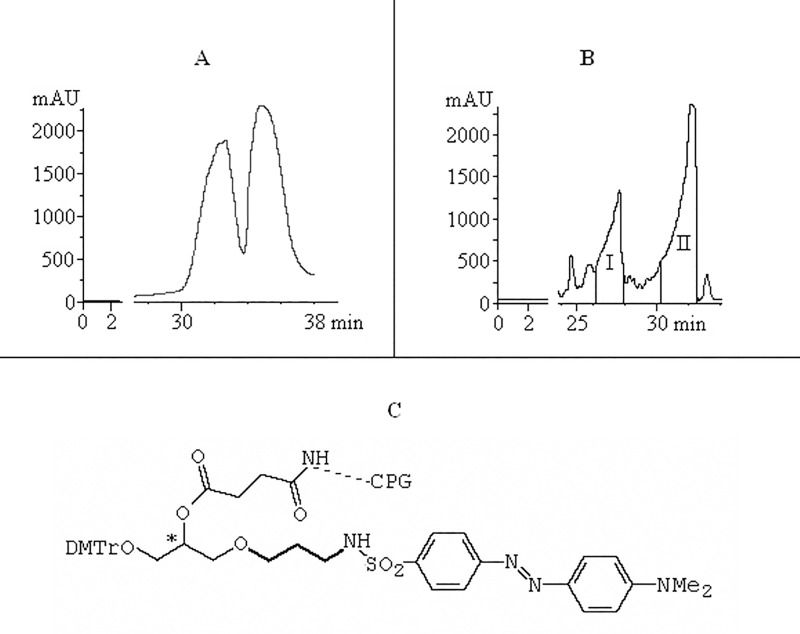
Dabsyl-derivatives. HPLC profiles of (A) 5'-FAM-27mer-Dabs-3' (F27D) and (B) 5'-DMTr-d(ACGT)_3_-Dabs-3' ODNs (12D). (C) The structure of Dabs-3'-CPG.

We suspected that similar difficulties may arise with H12D-ODN. The 3'-Dabsyl-residue in 3'-Dabsyl-CPG (Glen Research) has a racemic center (Fig 4C, *), and Dabs-ODNs sometimes give a double peak in HPLC profiles (see [24]); earlier we observed a double band in 5'-FAM-27mer-Dabs-3' (F27D) preparative HPLC profile (Fig 4A). It means that HEX-Dabs ODNs may exhibit up to four main bands on the HPLC profile after ammonolysis. We performed a trial experiment with non-fluorescent 5'-d(ACGT)_3_-Dabs-3'-ODN (12D). The preparative profile of its 5'-DMTr derivative contained two well-resolved peaks (Fig 4B, I and II) with 1:4 squares ratio. After DMTr group removal, the ODN from band (I) in contrast with the ODN from band (II) had molecular mass loss of -14 Da. We believe that a trimethylene bridge in Dabs-CPG (Fig 4C, marked with bold line) contained ~ 20% of dimethylene admixture. The presence of diastereomeric center in this case did not resulted in the peaks doubling, but to the asymmetric broadening of them.

**Fig 5:**
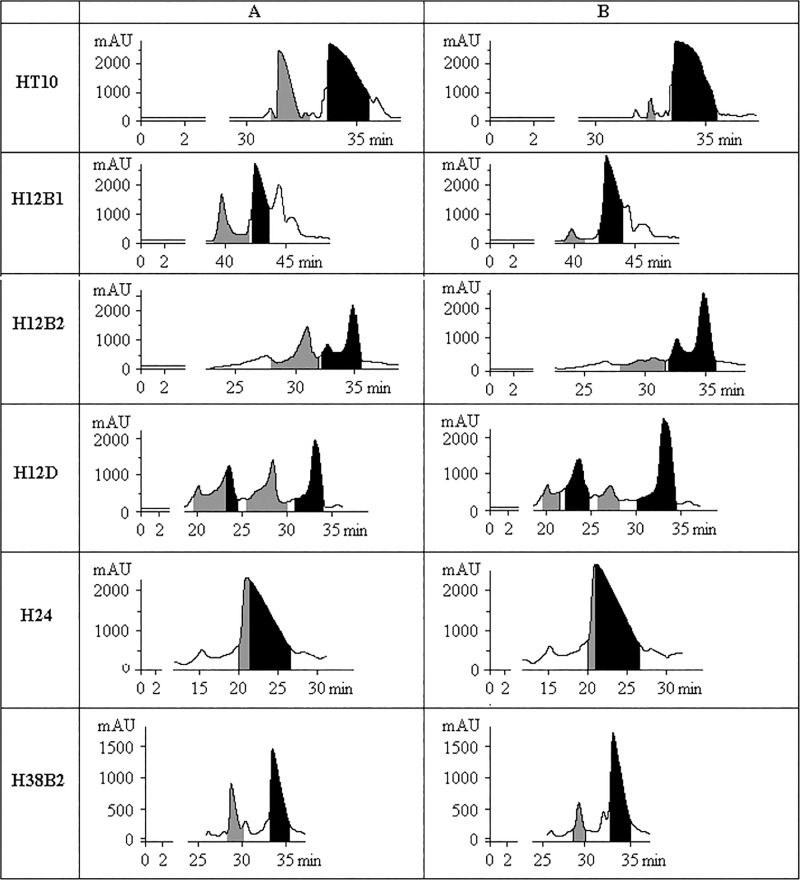
HPLC preparative profiles of several HEX-oligonucleotides. (A) After standard ammonolysis. (B) Obtained by new deblocking method. Grey filling up here and further marks **ACR-containing** zones and the black one corresponds to the **pure** HEX-ODNs fractions.

Three model HEX-12mer-QU-CPGs were subjected to ammonolysis as in [21], and after ammonia removal, the postsynthetic mixtures were separated with RP HPLC (Fig 5, profiles A). Fractions of each HEX-containing eluate were collected under permanent on-line monitoring of VIS-spectra, and the purity of the fractions was tested with analytical HPLC and MALDI-TOF mass-spectrometry.

It should be noted that practically no ACR-QU derivative, separated by HPLC from post-ammonolysis mixtures, was observed as a molecular specimen. Their preparative HPLC bands had spectral non-homogeneity, and additional signals with loss of tens of Da were found in their mass-spectra. This likely occurred because during automated oligonucleotide synthesis, the part of QU residues underwent damage of azo-groups and the defective HEX-QU-ODNs often have HPLC mobility similar to full-size ACR-QU-ODNs of the same sequences. These results likely indicate that these HPLC-purified ACR-probes are contaminated with 1–2 HEX-containing ODNs, which hinder the identification of ACR-derivatives by fluorescence. Therefore, we had to specially obtain pure ACR-QU-ODNs after extended ammonolysis (45°C/24 hrs, ~ 50% transformation) of HPLC-purified HEX-derivatives followed by re-chromatography. Instead of mixed H12B2, we used H38B2 for re-ammonolysis and the subsequent measurements. The first HEX-fraction of H12D (at approximately 23 min) was not spectrally pure and contained redundant peaks in the mass-spectrum, so we used the second (at approximately 34 min) HEX-Dabsyl fraction only.

**Fig 6:**
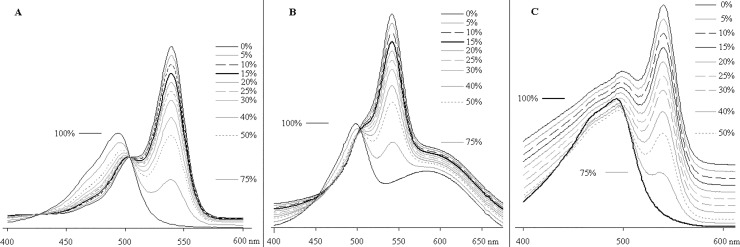
VIS-spectra of artificial mixtures of hexachloroflurescein-ODNs with different molar percentage of their hexachloroarylacridine analogs. (A) HEX-BHQ1. (B) HEX-BHQ2. (C) HEX-Dabs. 0.1 M ammonium acetate, pH 6.7, 10–20% MeCN, 45°C. Baselines of spectra are moved apart to separate the curves.

The spectral measurements of HEX-QU-ODNs with different quantities of ACR-QU-impurities show that quenchers' VIS spectra superimpose with the dye spectra and impede ACR determination when its content is lower than 15–20% for BHQ1-ODNs and 25–30% for BHQ2-derivatives. (Fig 6A–6C).

It became clear why ACR admixtures are not easily detectable in HEX-QU-probes. Optical data fail to reveal less than 15% of any ACR-QU-ODN. In mass-spectrometry, it is difficult to determine a -1 Da shift of molecular envelopes for ODNs with masses of several thousand Da. On electropherograms, the spots of HEX- and corresponding ACR-derivatives almost ever overlap and even coincide for quantities greater than 0.01 U per phoretic track (data not shown).

**Fig 7:**
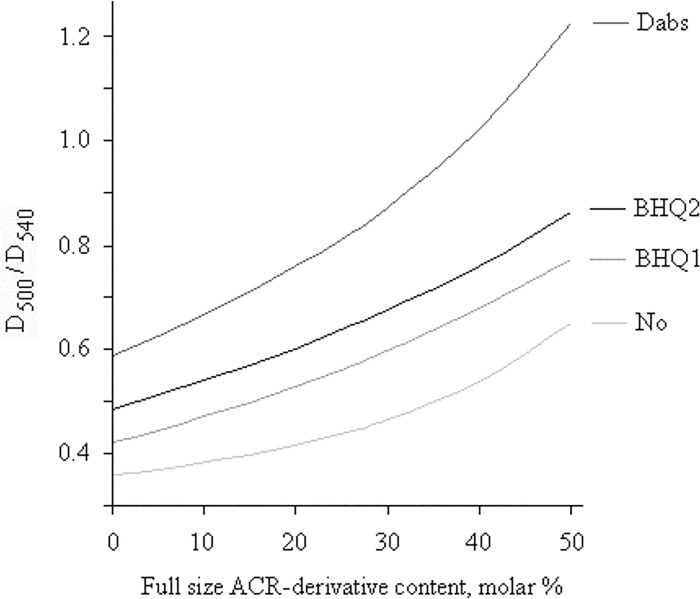
D_500_ / D_540_ ratios dependence from molar content of ACR-derivatives in HEX-oligonucleotides solutes. Conditions are as for Fig 6. The types of quencher are shown and the inscription "No" marks quencher-free ODN.

Thus, we are left with 1–2 sure methods: reverse phase HPLC in analytical mode under permanent control of eluate VIS spectrum, and maybe capillary electrophoresis as in [15]. Nevertheless, RP HPLC may sometimes define a presence but not a quantity of ACR-ODN in the sample. On the basis of obtained spectra, we evaluated D_500_ / D_540_ ratios and their dependence on the molar content of ACR-derivatives for HEX-, HEX-BHQ1-, HEX-BHQ2- and HEX-Dabs-ODNs (Fig 7), and we believe these curves to be useful for the quality evaluation of any HEX-ODN.

ACR admixtures significantly distort the curves of fluorescence accumulation (CFA) during PCR with artificial mixtures (Fig 2, compare curves A and B for H38B2 probe). Similar results were observed with another, H37B2-probe. We repeated the PCR trial with H38B2, obtained after ammonolysis followed by electrophoretic isolation (H38B2-e). Analytical HPLC of H38B2-e showed an expected 26% content of ACR-byproduct in this HEX-probe, and CFA recorded during PCR with this sample was practically congruent with curve B in Fig 2. This affirms that right acridine impurities deform the shapes of CFAs recorded with "quickly" purified HEX-probes.

Furthermore, we established that relatively short HEX-ODNs, with a length of less than 30 nucleotides, were more difficult to separate from ACR-byproducts with RP HPLC almost ever. The typical HPLC profiles are presented in Fig 5, H24 and H28B2 rows, column I. The peaks of ACR-derivatives and of target HEX-ODNs strongly overlapped, and the yields of pure ODNs were decreased significantly.

Therefore, we were faced with several reasons to develop a new method of HEX-ODNs deprotection that would ensure the lowest content of arylacridine byproducts.

### Optimization of HEX-oligonucleotide deblocking conditions

**Fig 8:**
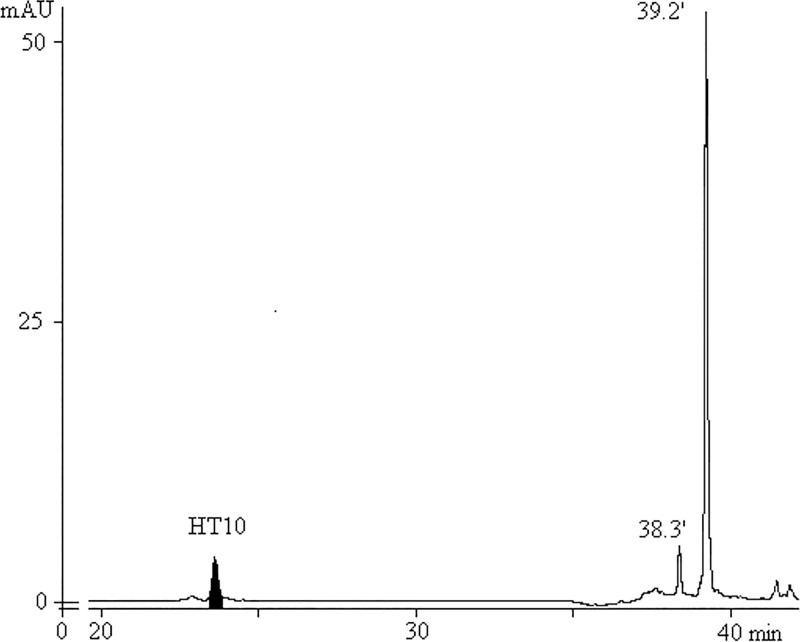
HPLC profile of HT10 ODN after K_2_CO_3_/MeOH deblocking, 24 hrs/room temperature.

To prevent the formation of ACR structures, we attempted to deblock HT10 ODN with potassium carbonate in methanol (24 hrs/room temperature), as a HEX-amidite manufacturer recommends (http://www.glenresearch.com/GlenReports/GR21-28.html, Table: Deprotection Conditions Suitable for Popular Dyes and Quenchers), but we obtained only a trace amount of target oligonucleotide because of poor removal of pivalic protection groups from the fluorophore. Prolongation of HT10 treatment up to 72 hrs resulted in approximately 3% of expected HT10 quantity. Fig 8 shows that, in accordance with mass-spectral data, the target peak is considerably weaker than byproduct peaks of monopivaloyl- (38.3') and dipivaloyl-derivatives (39.2'; HEX-amidite contains two pivaloyl groups protecting arylhydroxy groups on the fluorone cyclic structure).

**Fig 9:**
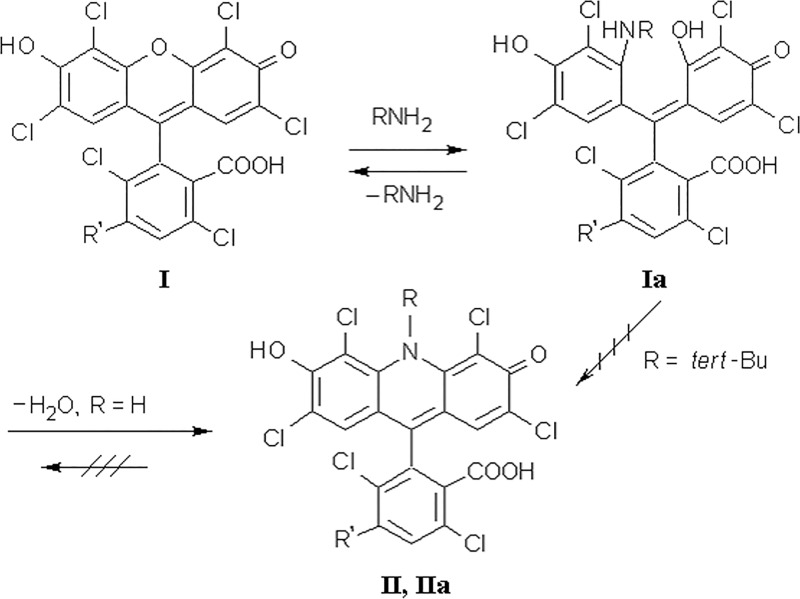
Supposed mechanism of ammonia and amines interaction with hexachlorofluorescein dye. R = 5'-ODN-spacer. R' = H (II), tert-Bu (IIa).

We then hypothesized that during the reaction of hexachlorofluorescein with a branched alkylamine, the recyclization of intermediate Ia into N-alkylacridine IIa (Fig 9, R = tert-butyl), would be sterically hindered and that the fluorone structure I would be re-formed again. We tested tert-butylamine as a potential deblocking agent because this strong base is easily accessible, water soluble and volatile.

Aqueous *tert*-butylamine application at moderate concentrations (15–30% v/v) and at room temperature for 24 hrs resulted in trace quantities of HT10. Increasing the *tert*-butylamine concentration or the temperature (up to 60°C) gave a post-deblocking mixture without ACR byproducts. However, the yield of pure HT10 ODN was even lower than after standard ammonolysis because a part of the pivaloyl groups was retained on the fluorophore. In addition, these solutes partially eliminated fluorophore from the target ODN. The deblocking of hetero-ODNs with aqueous *tert*-butylamine fared even worse. At previous conditions, a part of the pivalic group of 5'-HEX-d(ACGT)_4_−3' (H16) still was not removed completely from the HEX-residue. We varied *tert*-butylamine concentration, deblocking time, and temperature, but yields of H16 after preparative HPLC were not more than 50%, compared to the standard ammonolysis procedure. Moreover, the mass spectrum of purified H16 (5625 Da) contained additional signals, with the masses corresponding to mono- and di-isobutyryl derivatives (5691 and 5757 Da, respectively). This is likely due to some dG residues not being deblocked, and the obtained H16 ODN was of low quality. More stringent conditions caused appreciable modifications in the ODN chain.

It may be concluded that aqueous *tert*-butylamine fails to properly deblock HEX-ODNs and may cause the degradation of their sequences. Similar but less successful results were observed for the quencher-containing model probe 5'-HEX-d(ACGT)_4_−3'-BHQ2 (H16B) and for the more often used probes 5'-HEX-d(A_7_C_12_G_12_T_6_)-3'-BHQ2 (H37B2) and 5'-HEX-d(A_4_C_17_G_9_T_8_) -3'-BHQ2 (H38B2), whose HPLC profiles were additionally complicated with BHQ2-derivatives (data not shown). Nevertheless, we could summarize that in all these experiments, no formation of acridines was registered and that *tert*-butylamine must be used only at room temperature, but it is too weak to reliably remove branched protection groups.

We further tested different mixtures of aqueous ammonia with *tert*-butylamine as potential deblocking agents (21–24 hrs/ambient temperature).

After these series of experiments, we discovered that the addition of a 15–30% (v/v) of *tert*-butylamine to aqueous ammonia prevents the formation of acridines, guarantees complete elimination of all protecting groups and, as a result, increases the yields of pure target hexachlorofluorescein-labeled ODNs by at least 25%. The yields of each single HEX-ODN were almost equal in this gamut of *tert*-butylamine content in ammonia. Reliable lowering of yields of probe H38B2, for example, was observed for mixtures with *tert*-butylamine concentration >35%. This observation is valid for all tested HEX-, HEX-BHQ1-, HEX-BHQ2- and HEX-Dabs-labeled ODNs (data not shown). The deblocking of the three mentioned model HEX-12mers and of the "short" H24 and H28B2 ODNs by aqueous ammonia—*tert*-butylamine mixtures also yielded good results (Fig 5, column B), as well as for the assemblage of HEX-ODNs listed in S2 Table. Data in the fourth column with the title beginning "Enhancement of the yield…" (S2 Table) were averaged from ≥4 samples after contemporaneous syntheses on the multichannel DNA-synthesizer. The less difference there was between the analytical HPLC retention times between HEX- and ACR-derivatives, the greater the benefit was from the new deblocking method.

**Fig 10:**
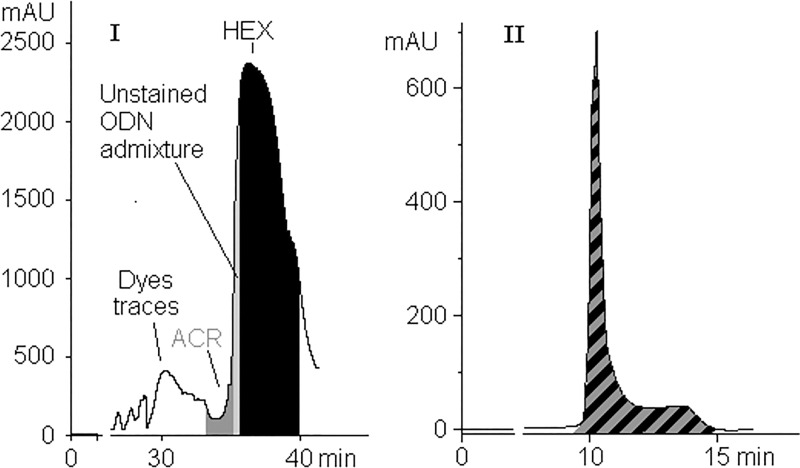
HPLC profiles of H50 ODN. (A) Preparative profile after our debloking method. (B) Analytic HPLC of pure H50 after its re-ammonolysis.

The example of H50 ODN (5'-HEX-d(C_25_T_25_)-3') is especially remarkable. ODNs containing oligo-dC framents easily form intermolecular aggregates in acidic solutes due to formation of so-called *i*-motifs [24]. We successfully purified H50 after using the new deblocking method and noted that the retention time of H50 was not routine for similar HEX-ODNs and we obviously dealt with the composition of aggregations, which were already quite stable at practically neutral pH values. After re-ammonolysis of pure H50, it was impossible to separate HEX- and ACR-derivatives of d(C_25_T_25_), even under analytical conditions, and it was clear that we could not obtain pure H50 after the routine deblocking procedure (see Fig 10).

All HEX-derivatives obtained with tert-butylamine—aqueous ammonia mixtures were tested by UV-VIS spectroscopy (with the calculation of D_500_ / D_540_ ratios), mass-spectroscopy, RP HPLC in analytical mode, as well as by real-time PCR with H37B2 and H38B2 probes.

The mass-spectra of all these HPLC-isolated HEX-ODNs do not contain additional peaks and confirm the absence of modifications in ODN sequences. The spectrum of mixed probe H12B2 contains two expected signals with masses corresponding to 5'-HEX-d((ACGT)_2_-ACGG)-BHQ2 and 5'-HEX-d(ACGT)_3_-BHQ2 structures. Careful HPLC (C18 and C4 reverse phase columns, see S2 Table) qualifying under on-line VIS-spectrum monitoring showed single peaks of isolated probes without arylacridine traces.

CFAs from RTPCRs performed with H38B2 ODN obtained by the standard preparation procedure and by the new method were tightly similar and finally confirmed high quality of newly gained probe.

We have to note that the majority of HEX-BHQ2 probes obtained via our new method and then isolated by electrophoresis only, resulted in approximately 5% more flat CFAs than after HPLC-purification and were quite suitable for the screening of the most effective probe. See S1 Fig, where analytic profiles of H23B2 ODN after different method of deprotection and electrophoretic isolation are compared.

This new method for obtaining hexachlorofluorescein-labeled oligodeoxynucleotides has been protected with a patent [25].

### Photochemical destruction of hexachloroarylacridine

**Fig 11:**
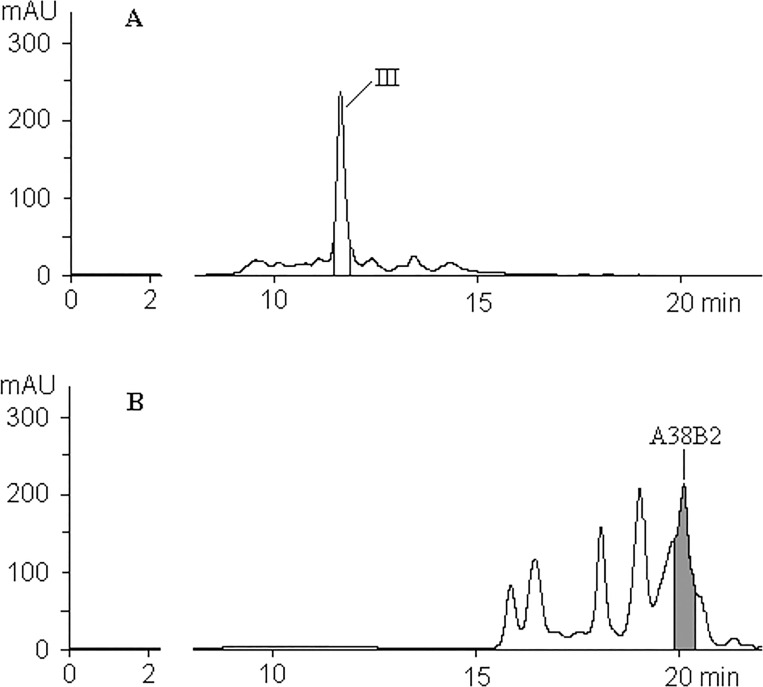
HPLC profiles of A38B2 after photochemical transformation. Incubation: (A) 1 hour in conc. NH_4_OH under the daylight; "18-HQ"-gradient. The structure of diamino-derivative (III) see in Fig 1. (B) - 25 minutes under PCR-mimic conditions, 64°C; HPLC gradient: 8–60% B-buffer / 25 min. Colourless peaks correspond to non-fluorescent derivatives.

A last question remains to be answered about the noticeable difference between CFAs with HEX- and ACR-probes. When re-ammonolyses of pure HEX-probes were performed in UV-transparent tubes, the yields of target ACR-ODNs were very unexpectedly low. We supposed the tetrachloroacrydine cycle was disrupted via photochemical process with some nucleophilic agents and incubated pure A38B2 probe in concentrated ammonia at ambient temperature under daylight. After 1 hour, analytical HPLC showed no fluorescent component present in the solution and achieved 50% molar content of the new BHQ2-derivative with a molecular mass of 12820 Da, which was 17 Da more than the initial A38B2 (Fig 11A). We believe that the photochemical disruption of the acridine cycle took place with production of diamino-derivative (Fig 1, III). Furthermore, we are convinced that under PCR-like conditions (PCR-buffer, 64°C, λ_ex_ = 530 nm, λ_em_ = 565 nm) the ratio of fluorescence values between equimolar solutions of HT10 and AT10 remained at 8:1. The final experiment consisted of incubation of A38B2, the acridine analog of the H38B2 probe, in a full-component PCR-solution without nucleotide-triphosphates or polymerase in a quartz cuvette under wideband UV-irradiation. After 25 minutes (from this time the noticeable accumulation of fluorescence begins, see Fig 2) of irradiation, only ~20% of A38B2 ODN was retained in the mixture (Fig 11B). We did not investigate the nature of the byproducts. It is sufficient that 80% of the arylacrydine ODN in the PCR process loses fluorescent properties, and this likely explains why CFAs obtained with arylacridine probes are so flat.

Finally, it should be noted that adding *tert*-butylamine to ammonia solution does not complicate the post-deblocking procedures.

In this study, we have reported a novel, simple approach for postsynthetic deblocking of hexachlorofluorescein-labeled oligonucleotides.

## Conclusions

Post-ammonolysis mixture of any hexachlorofluorescein-labeled oligodeoxyribonucleotide contains more than 25% of full-sized hexachloroarylacridine derivative. Acridine byproduct is not easily detectable or removable, and it may significantly distort the results of fluorescence quantitative analyses with hexachlorofluorescein probes.

The optical properties of hexachloroarylacridine oligonucleotides and their mixtures with hexachlorofluorescein analogs were established.

Hexachloroarylacridine fluorophore, under PCR-like mimetic conditions, is significantly transformed into non-fluorescent derivatives.

The new, simple, and reliable method of postsynthetic deblocking of hexachlorofluorescein-labeled oligonucleotides is designed to allow these fluorescent probes to be obtained at high yields and with the guaranteed absence of acridine byproducts.

## Supporting Information

S1 Fig Analytic HPLC profiles of H23B2 probe. A–after ammonolysis, B—after ammonolysis and electrophoresis, C—after deblocking with 15% *tert*-butylamine in ammonia and the following electrophoresis (D). 18HQ HPLC method
(TIF)

S1 Table Use and the methods of purification of HEX-probes. HEX–hexachlorofluorescein, RP HPLC–reverse phase HPLC, QU—quencher, EF– electrophoresis, Dabs—Dabsyl, IBHQ—Iowa Black Hole Quencher.
(DOC)

S2 Table List of the oligodeoxyribonucleotides used in this paper. Letter A in the name of ODN means ACR-residue, B1 –BHQ1-, B2 –BHQ2-, D–Dabs-, F–FAM-, and H–HEX-residues. The conditions of routine analytical HPLC are described in the MATERIALS AND METHODS section. * Empty cells in this column indicate that only the new method was applied. ** 21–27% B / 30 min, 45°C. *** Preparative gradient for F27D: C4 column, 8–24% B / 40 min, 45°C.
(DOC)

## Abbreviations

ODN: oligodeoxyribonucleotide
HEX: hexachlorofluorescein
RTPCR: real-time PCR
QU: quencher
ACR: hexachloroarylacridine
RP HPLC: reverse phase HPLC
Dabs: Dabsyl
CPG: Control Pore Glass
BHQ-1: Black Hole 1
BHQ-2: Black Hole 2
CFA: curve of fluorescence accumulation
