# Supplementary figures and images for: A Solution to the Common Problem of the Synthesis and Applications of Hexachlorofluorescein Labeled Oligonucleotides
Source: PLoS One. 2016 Nov 18;11(11):e0166911. doi: 10.1371/journal.pone.0166911 (PMC5115841; doi:10.1371/journal.pone.0166911)

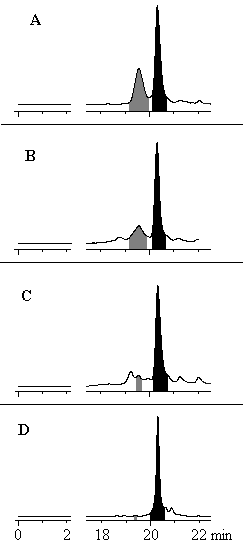

Supplement: S1 Fig — A–after ammonolysis, B—after ammonolysis and electrophoresis, C—after deblocking with 15% tert-butylamine in ammonia and the following electrophoresis (D). 18HQ HPLC method (TIF) [file pone.0166911.s001.tif]
