# Supplementary material for: A Solution to the Common Problem of the Synthesis and Applications of Hexachlorofluorescein Labeled Oligonucleotides
Source: PLoS One. 2016 Nov 18;11(11):e0166911. doi: 10.1371/journal.pone.0166911 (PMC5115841; doi:10.1371/journal.pone.0166911)
Supplement: S1 Table — HEX–hexachlorofluorescein, RP HPLC–reverse phase HPLC, QU—quencher, EF– electrophoresis, Dabs—Dabsyl, IBHQ—Iowa Black Hole Quencher. (DOC) [file pone.0166911.s002.doc]

**S1 Table**.Use and the methods of purification of HEX-probes.

| Bulk content of probes, reference | What was estimated | Measured parameter of HEX-probe or used method | Probe purification method (if described) | Probe quality verification method |
| --- | --- | --- | --- | --- |
| 5'-HEX-20-mer [2] | Delivery of ODNs to the living cells | Cells fluorescence | Purchased | No |
| 5'-HEX-25-mer, containing phosphorothioate bonds [3] | ODN delivery to mouse retinal cells | Fluorescence microscopy | Purified by supplier | No |
| 5'-HEX-22-mer [4] | Mechanism of endonuclease cleavage of triplexes | Fluorescence intensity | RP HPLC | Not shown |
| 5'-HEX-35-mer [5] | Movement of bipedal DNA walker | Multiplexed fluorescence quenching | Purified by supplier | No |
| 5'-HEX-15-mer [6] | Polymer-DNA sensor properties | Shift in electrophoretic mobility, changes in UV and fluorescence spectra | Purchased | No |
| 5'-HEX-16-mers [7] | Catalytic mechanism of endonuclease | Percent of probe cleavage | RP HPLC | PAAG EF |
| 5'-HEX-31-mer [8] | Mechanism of ATP-dependent helicase activity | Fluorescence intensity | PAAG EF | Not shown |
| 5'-HEX-21-mer [9] | Protein-DNA interaction | Fluorescence anisotropy | Not shown | Not shown |
| 5'-HEX-16-mers [10] | Fluorescence resonance energy transfer between FAM- and HEX-dyes | Changes in fluorescence spectra | PAAG EF | No |
| HEX-22-mer-NH_2_ -3' [11] | Detection of hot spot mutations | Melting curve analysis | Purchased | Not shown |
| 5'-HEX-21-mer [12] | Detection of several genes | Multiplex RTPCR | Purchased | No |
| 5'-HEX-20- and 24-mer-tetramethyl-rhodamine-3' [13] | Comparison of siRNA delivery methods | Tissue distribution analysis with QPCR | Purchased | Not shown |
| 5'-HEX-32-mers-Dabs-3' [14] | Simultaneous detection of *Legionella* species | Multiplex RTPCR | Purchased | Not shown |
| 5'-HEX-20- and 21-mer [15] | Genotyping | Multicolor multiplex PCR | capillary  EF | Not shown |
| 5'-HEX-36 -- 42-mers-IBHQ-3' [16] | Molecular beacon properties | Multiplexed DNA detection, fluorescence  microscopy | Purchased | No |
| 5'-HEX-25-mer-IBHQ-3' [17] | Detection of several raphidophyte species | Quantitative PCR | Not shown | Not shown |
| 5'-HEX-26-mer-Dabs-3' [18] | Classification of gene variants | Multiplex PCR | RP HPLC | Not shown |
| 5'-HEX-oligomers-Dabs-3' [19] | Simultaneous detection of virus types | Multicolor PCR | Purchased | No |
| 5'-HEX-11- and 20-mer and 5'-HEX-11- and 20-mer-3'-QU [20] | Duplex stability-dependence on the types of fluorescent dye and quencher | Fluorescence and UV melting | PAAG EF, ion pairing HPLC, ion-exchange HPLC | Mass- spectro-metry, capillary EF |

HEX – hexachlorofluorescein, RP HPLC – reverse phase HPLC, QU - quencher, EF – electrophoresis, Dabs - Dabsyl, IBHQ - Iowa Black Hole Quencher.
