# Supplementary material for: A Solution to the Common Problem of the Synthesis and Applications of Hexachlorofluorescein Labeled Oligonucleotides
Source: PLoS One. 2016 Nov 18;11(11):e0166911. doi: 10.1371/journal.pone.0166911 (PMC5115841; doi:10.1371/journal.pone.0166911)
Supplement: S2 Table — Letter A in the name of ODN means ACR-residue, B1 –BHQ1-, B2 –BHQ2-, D–Dabs-, F–FAM-, and H–HEX-residues. The conditions of routine analytical HPLC are described in the MATERIALS AND METHODS section. * Empty cells in this column indicate that only the new method was applied. ** 21–27% B / 30 min, 45°C. *** Preparative gradient for F27D: C4 column, 8–24% B / 40 min, 45°C. (DOC) [file pone.0166911.s003.doc]

**S2 Table. List of the oligodeoxyribonucleotides used in this paper.**

| The name of ODN | ODN content without dyes | Analytic HPLC data: retention times (min) and conditions | Enhancement of the yeild of HPLC pured ODN after the change of standard ammonolysis with new deblocking method * |
| --- | --- | --- | --- |
| HT10  AT10 | T_10_ | 16.2**  15.6** | 26% |
| H38B2  A38B2 | d(A_4_C_17_G_9_T_8_) | 12.2 (18-HQ), 12.1 (4-HQ)  10.6 (18-HQ), 10.6 (4-HQ) | 35% |
| H37B2  A37B2 | d(A_7_C_12_G_12_T_6_) | 13.7 (18-HQ), 12.4 (4-HQ)  12.1 (18-HQ), 11.0 (4-HQ) | 36% |
| H37'B2  A37'B2 | d(A_8_C_12_G_13_T_4_) | 13.0 (4-HQ)  11.4 (4-HQ) | 30% |
| H12  A12 | d(ACGT)_3_ | 11.8 (18H)  10.3 (18H) | 31% |
| H12B1  A12B1 | d(ACGT)_3_ | 21.5 (18-HQ)  20.5 (18-HQ) | 28% |
| H12B2  A12B2 | d(ACGT)_3_  d[(ACGT)_2_ACCT]  d(ACGT)_3_  d[(ACGT)_2_ACCT] | 18.0 (18-HQ), 17.3 (4-HQ)  15.9 (18-HQ), 15.8 (4-HQ)  17.4 (18-HQ), 17.2 (4-HQ)  14.2 (18-HQ), 15.2 (4-HQ) | 38%  32% |
| F27D | d(A_8_C_11_G_5_T_3_) | 32.4 + 34.7, ~1:1 squares ratio, preparative gradient *** | after standard ammonolysis only |
| 12D | d(ACGT)_3_ | 13.5 + 18.8 (18-HQ), 1:4 squares ratio | after standard ammonolysis only |
| H12D  A12D | d(ACGT)_3_ | 10.7 (18-HQ)  11.8 (18-HQ) | 33% |
| H21B2  A21B2 | d(A_3_C_7_G_6_T_5_) | 15.4 (18-HQ)  14.9 (18-HQ) | 54% |
| H38B2  A38B2 | d(A_8_C_11_G_7_T_2_) | 16.0 (18-HQ)  14.9 (18-HQ) | 36% |
| H25B2  A25B2 | d(A_6_C_7_G_7_T_5_) | 18.3 (18-HQ)  17.5 (18-HQ) | 41% |
| H23B2  A23B2 | d(A_4_C_11_G_4_T_4_) | 18.3 (18-HQ)  17.5 (18-HQ) | 52% |
| H22B2  A22B2 | d(A_3_C_7_G_2_T_10_) | 20.5 (18-HQ) 17.3 (4-HQ)  20.0 (18-HQ) 16.6 (4-HQ) | 55% |
| H24  A24 | d(A_7_C_4_G_7_T_6_) | 9.3 (18-H)  9.0 (18-H) | 59% |
| H19B2  A19B2 | d(A_3_C_7_G_2_T_10_) | 13.9 (4-HQ)  13.7 (4-HQ) | 60% |
| H20B2  A20B2 | d(A_1_G_6_C_7_T_6_) | 16.2 (18-HQ) 7.5 (4-HQ)  14.8 (18-HQ) 6.6 (4-HQ) |  |
| H21B2  A21B2 | d(A_5_C_8_G_3_T_5_) | 23.1 (18-HQ) 15.4 (4-HQ)  22.6 (18-HQ) 15.2 (4-HQ) | 53% |
| H22B2  A22B2 | d(A_2_C_8_G_7_T_5_) | 18.4 (18-HQ) 13.1 (4-HQ)  17.1 (18-HQ) 12.1 (4-HQ) | 43% |
| H22'B2  A22'B2 | d(A_3_C_7_G_2_T_10_) | 13.9 (4-HQ)  13.7 (4-HQ) |  |
| H23B2  A23B2 | d(A_8_C_6_G_8_T_1_) | 20.3 (18-HQ) 12.0 (4-HQ)  19.6 (18-HQ) 12.5 (4-HQ) | 39% |
| H24B2  A24B2 | d(A_7_C_10_G_5_T_2_) | 14.8 (18-HQ) 7.0 (4-HQ)  13.7 (18-HQ) 6.9 (4-HQ) | 35% |
| H24'B2  A24'B2 | d(A_3_C_9_G_7_T_5_) | 19.5 (18-HQ) 11.7 (4-HQ)  18.9 (18-HQ) 11.2 (4-HQ) |  |
| H26B2  A26B2 | d(A_9_C_9_G_5_T_3_) | 20.1 (18-HQ) 13.1 (4-HQ)  19.5 (18-HQ) 12.8 (4-HQ) |  |
| H28B2  A28B2 | d(A_7_C_9_G_6_T_6_) | 18.6 (18-HQ) 10.8 (4-HQ)  18.0 (18-HQ) 10.4 (4-HQ) | 48% |
| H28'B2  A28'B2 | d(A_10_C_10_G_5_T_3_) | 19.8 (18-HQ) 12.1 (4-HQ)  19.3 (18-HQ) 11.9 (4-HQ) |  |
| H30B2  A30B2 | d(A_7_C_6_G_9_T_8_) | 16.5 (18-HQ) 9.3 (4-HQ)  15.7 (18-HQ) 8.7 (4-HQ) | 40% |
| H50+A50 | d(C_25_T_25_) | 10.2 (4-HQ) not resolved |  |

Letter A in the name of ODN means ACR-residue, B1 – BHQ1-, B2 – BHQ2-,

D – Dabs-, F – FAM-, and H – HEX-residues. The conditions of routine analytical HPLC are described in the MATERIALS AND METHODS section.

* Empty cells in this column indicate that only the new method was applied.

** 21-27% B / 30 min, 45°C.

*** Preparative gradient for F27D: C4 column, 8-24% B / 40 min, 45°C.
